# Back-to-School Screening for Children with Cancer and Hematologic Disorders: Bridging Healthcare and Education

**DOI:** 10.5334/cie.288

**Published:** 2026-01-29

**Authors:** Michelle Fritsch, Ashley Matthews, Mashal Kara, Anastasia Deeter

**Affiliations:** 1 Texas Children’s Hospital, Baylor College of Medicine, US; 2 Texas Children’s Hospital, US

**Keywords:** Chronic illness, pediatric oncology, hematology, academic performance, educational intervention, resources, healthcare professionals, siblings, hospital school program, community engagement, school re-entry

## Abstract

Children with pediatric chronic illnesses, including cancer and hematologic disorders, face psychosocial challenges that disrupt development, academic progress, and social functioning. While healthcare providers often connect families to supportive resources, comprehensive approaches to school-related needs remain limited. The Back-to-School Screening Program, a practice-based intervention evaluated using a descriptive mixed-methods design, was developed to systematically identify the educational needs of children with cancer and blood disorders at the start of the academic year. The evaluation combined quantitative data from psychosocial screening surveys and service utilization metrics with qualitative feedback from families and interdisciplinary staff. Launched during the COVID-19 pandemic, the program assessed available psychosocial resources, identified barriers to school participation, and facilitated communication between healthcare and educational systems. Between 2020 and 2024, 1,193 families were screened. Results showed measurable improvements in educational preparedness, family satisfaction, and cross-sector collaboration. Outcomes included increased access to educational materials and technology, enhanced family understanding of school supports, and strengthened coordination among healthcare, school, and community partners. Findings highlight the value of embedding educational screening within pediatric psychosocial care to prevent academic disruption and promote resilience. The Back-to-School Screening Program provides a replicable model for integrating educational advocacy into routine care, informing both practice and policy to support the long-term wellbeing of children with chronic illness.

Children undergoing treatment for cancer and hematologic disorders experience significant disruptions to their educational engagement and psychosocial development (Cantrell & Kelly, 2015; Wiener et al., 2015). Hospitalization, treatment-related side effects, and frequent school absences interfere with academic progress as well as social connectedness and emotional wellbeing (Emerson et al., 2016; Hocking et al., 2018). For many families, navigating educational systems during treatment adds substantial stress to already complex medical demands (Thompson et al., 2015). These challenges were further intensified during the COVID-19 pandemic, which amplified caregiver strain particularly for mothers balancing employment, caregiving, and educational responsibilities amid widespread school disruptions (Heggeness, 2020). Although school participation is increasingly recognized as an essential component of holistic pediatric care, most hospital-based programs rely on individualized support or school re-entry assistance rather than standardized approaches to identify educational barriers early in treatment, resulting in delayed identification of needs, inconsistent supports, and inequities in access to educational accommodations, outcome that mirror national patterns documented in early attendance and chronic absenteeism research (Attendance Works & Healthy Schools Campaign, 2015). The absence of systematic, healthcare-embedded screening for school-related needs at predictable transition points, therefore, represents a critical gap in current practice (Irwin et al., 2024).

The Back-to-School Screening Program was developed to proactively assess educational needs at the beginning of each academic year for children diagnosed with cancer and blood disorders. The initiative was designed as a systematic, team-based approach to bridge communication between healthcare providers, educators, and families (Wiener et al., 2015), and thereby ensure that learning and developmental goals remain central throughout the course of medical care. Further, by integrating screening practices within psychosocial care, the program aims to promote early identification of educational barriers, enhance collaboration between disciplines. This paper describes the development, implementation, and outcomes of this practice-based and scalable screening model, which seeks to close the gap between healthcare and education for children with chronic illness.

## Literature Review on Existing Education Screening Initiatives

To contextualize the development of this program, the following review examines educational support and screening approaches within pediatric and hospital-based care.

Much of the existing research highlights the significant challenges pediatric patients face in maintaining educational continuity during treatment, including the cumulative academic risks associated with chronic absenteeism. (Forrest et al., 2011; Jacob & Lovett, 2017; U.S. Department of Education, 2025a). For example, studies consistently emphasize the cognitive, emotional, and logistical barriers associated with long-term hospitalization and treatment, including concentration difficulties, changes in stamina, or reduced mobility (Hocking et al., 2018; Thompson et al., 2015). In comparison, there is evidence supporting the benefits of integrated school support services and hospital-based education programs that maintain learning engagement and social connection during hospitalization and active treatment (Peterson et al., 2005; Thompson et al., 2015).

Nevertheless, the literature review revealed limited research focused specifically on tools or systematic procedures for screening school-related needs within pediatric healthcare settings. While numerous studies advocate for multidisciplinary coordination and structured reintegration programs (Hocking et al., 2018; Thompson et al., 2015), few describe validated instruments or frameworks to identify educational barriers in the medical journey. Existing program initiatives tend to rely on reactive referrals rather than standardized assessments embedded in healthcare workflows (Delloso et al., 2021). This lack of structured, evidence-informed screening mechanism represents a critical gap in practice. Recent literature has begun to address aspects of school re-entry and educational continuity for children with chronic and life-threatening illnesses, yet the approaches vary in design and implementation. Some studies emphasize the importance of collaboration between teachers, families, and hospital teams in supporting academic reintegration for students with cancer (Delloso et al., 2021; Irwin et al., 2024). Similarly, Thompson et al., 2015; Delloso et al., 2021, describe systemic models of school re-entry that propose instructional frameworks to address policy and communication barriers between educational and healthcare sectors. These studies demonstrate the need for coordinated cross-sector approaches; however, they differ in operational focus and do not consistently incorporate structured, healthcare-embedded screening mechanisms capable of identifying educational needs.

Additional challenges arise from misalignment between healthcare and educational systems. School personnel may have limited understanding of the medical, cognitive, and psychosocial implications of ongoing treatment, while healthcare providers may lack familiarity with educational policies, school-based services, and legal protections relevant to students with chronic illness (Hocking et al., 2018; Thompson et al., 2015; U.S. Department of Education, 2025b). These gaps highlight the need for a coordinated framework that supports shared understanding and communication across settings.

Internal data, including consultations, evaluations, and family surveys, corroborate findings in the literature. Families frequently report uncertainty about how to maintain schooling during treatment and limited understanding of available supports (Hocking et al., 2018). School staff members expressed a need for better communication and clearer transition planning when students return to school. These internal observations mirror patterns described studies of teacher perspectives on school re-entry programs (Irwin et al., 2024), parent experiences of maintaining school during treatment (Delloso et al., 2021), and models addressing educational continuity in chronic illness. Taken together, the evidence demonstrates a substantial opportunity to embed systematic screening for educational barriers within pediatric healthcare. The current published and internal findings highlight the absence of standardized procedures to identify learning needs and coordinate appropriate interventions across settings.

## A Purpose of the Study/Intervention

Building upon these identified gaps, the Back-to-School Screening Program was developed as practice-based intervention designed to (a) systematically assess academic, psychosocial, and resource needs; (b) connect families to appropriate educational and community supports; and (c) ensure that children undergoing treatment can maintain engagement in learning and achieve key academic milestones. The program’s methodological framework integrates descriptive and process evaluation components to assess feasibility, implementation fidelity, and family-reported outcomes to address the lack of systemic, healthcare embedded screening mechanism identified in the literature.

## Methods

A descriptive, mixed-methods process evaluation within a practice-based intervention design was used to assess the implementation, feasibility, and outcomes of the Back-to School Screening Program. The mixed-methods approach combined quantitative data (screening frequencies, identified needs, referral rates, and participation metrics) and qualitative data (semi-structured family interviews and staff reflections). This framework was selected to capture both the measurable program outcomes and contextual factors influencing implementation, consistent with process evaluation methodologies in similar studies (Kent et al., 2019).

## Program Design/Setting

The Back-to-School Screening Program was implemented across inpatient and outpatient settings, with screenings conducted annually in the month preceding each academic year. Screenings focused on identifying academic, psychosocial, and resource-related needs and facilitating coordination among families, healthcare teams, and schools. Launched in 2020, the initiative evolved during the COVID-19 pandemic to incorporate hybrid and remote learning considerations, digital tools, and community-based resource in response to increased family vulnerability, disruption of routines, and heightened risk for child psychosocial difficulties (Fosco et al., 2022).

## Participants/Sample

Eligible participants included pediatric patients aged 3 to 18 years old with cancer or blood disorders who were actively receiving treatment or follow-up care at the participating institution, along with their families. Participation was voluntary. Families who declined participation or were unavailable during the screening period were excluded from the study. In 2020, screening was offered in the cancer and hematology center from July 1-August 31. From 2021 onward screening occurred annually during a designated two-week period in July. Recruitment took place through interactions with psychosocial staff during inpatient and outpatient encounters.

## Screening Tool Description and Development

The Back-to-School Screening Tool was co-developed by a multidisciplinary psychosocial team, including child life specialists, school coordinators, and social workers, with input from nursing and patient/family assistants (Appendix 1). Tool development was informed by a review of existing literature and school-based screeners, as well as informal feedback from families and staff. The tool underwent pilot testing and ongoing refinement through biweekly team meetings. Sample items are included in Appendix 1, and the instrument’s structure and scoring approach are described in the Results section. Although the tool was not formally validated, face validity was established through expert review and pilot implementation during the 2020 rollout.

## Structure and Domains

The tool assessed four domains: academic functioning, technology access, psychosocial support, and resource needs. Responses were collected using a structured form with both categorical (yes/no, Likert-type) and open-ended items. A sample version of the tool is provided in Appendix 2.

## Data-Collection Procedures

Psychosocial and school coordinators conducted screenings using standardized scripts, either in person or by phone based on family availability. In addition, child life specialists and social workers supported data collection and follow-up as needed. Screening data were aggregated and reviewed by the multidisciplinary psychosocial team to inform referrals and program planning.

Data sources included completed screening forms, family interviews, staff feedback, and chart documentation. Needs were defined as gaps or barriers related to academic accommodations, technology access, psychosocial wellbeing, or material resources (e.g., financial, nutritional, or transportation needs).

Outcome measures included:

- Quantitative: Frequencies and percentages of families reporting each need category; number and type of referral generated (Table 2).
- Qualitative: Thematic coding of open-ended responses regarding family satisfaction, perceived preparedness for school re-entry, and barriers encountered.
- Process Indicators: Participation rate, staff engagement, and implementation fidelity to planned procedures.

## Data Analysis

Quantitative data were summarized using descriptive statistics (frequencies, percentages, and year-to-year comparisons). Qualitative data from open-ended responses and staff debriefings underwent thematic analysis using an inductive coding approach. Data were coded for recurring patterns; discrepancies were resolved through consensus. Emerging themes were grouped under three analytic categories: (a) family experience, (b) systematic barriers, and (c) program improvement opportunities. Results were synthesized to evaluate program reach, relevance, and opportunities for enhancement in connecting families to educational resources.

## Ethical Approval and Consent

Ethical approval was obtained through the participating hospital’s institutional leadership. Families were provided with verbal and written information outlining the program’s purpose, voluntary nature, and confidentiality protections. Verbal consent was obtained prior to participation; all data were de-identified and analyzed in aggregate. Participation or non-participation did not affect clinical care.

## Community Partnerships and Resource Integration

To enhance reach and sustainability, the program developed partnerships with established local community organizations in providing school supplies, internet access, food assistance, and dental/vision services. Resource materials were distributed in English and Spanish. These collaborations extended program benefits to families with heightened social vulnerabilities and strengthened equity across patient population.

## Implementation Timeline

Program planning commenced four months before initial rollout (see Appendix 1), with milestones for staff training, leadership approval, and coordination across care units. Major phases include:

1. 2020: Initial design and pilot implementation
2. 2021: Introduction of school coordinators
3. 2022–2024: Integration of community partnerships and digital tracking of outcomes
4. 2025: One-day Back-to-School event

This phased approach allowed for iterative refinement of the screening tool and program evaluation procedures, ensuring alignment with evolving educational and healthcare needs.

## Outcomes/Results

From 2020 through 2024, the Back-to-School Screening Program screened and supported a total of 1,193 families of children with cancer and blood disorders. The data, in Table 1, reflect steady program growth, increased refinement of screening processes, and evolving family needs over time. Since its establishment (2020), the Back-to-School Screening Program staff has consistently met its goal of promoting academic continuity and addressing barriers to education for children undergoing medical treatment.

**Table 1:** Technology, Support Services, and Key Contacts by School District 2021.


DISTRICT	HOTSPOT AVAILABILITY	TECHNOLOGY ASSISTANCE	OTHER ASSISTANCE/PROGRAMS	CONTACT PERSON DETAILS

Independent School District	Yes	Yes – pick up on Aug 14	Free lunch	S Smith 111-111-1111


## Quantitative Findings

Between 2020 and 2024, families received a range of educational supports, including school supplies, technology devices, and referrals to community-based resources. The proportion of families receiving backpacks and school supplies increased steadily each year, demonstrating the program’s growing capacity and sustaining partnerships with community donors (Table 2). Similarly, the provision of computers and tablets expanded significantly during and after the COVID-19 pandemic, addressing digital inequities in learning environments (Vogels et al., 2020).

**Table 2:** Educational and Psychosocial Support Outcomes: Data Trends and Interpretation 2020–2025.


YEAR	2020	2021	2022	2023	2024	2025	DATA INTERPRETATION

**Families Screened**	318	310	235	190	140	200	The reduction reflects changes in the scope of the screening process following the addition of three school coordinators, two focused on educational advocacy and one providing support within the hospital.

**Backpacks/School Supplies Provided**	100	395	425	420	500	409	The increase indicates a growing demand for these resources.

**Social Work Needs**	43	33	4	11	8	On-site assistance	The decrease is likely due to a reduced demand after the initial pandemic period when hybrid and remote learning support was more critical.

**Computer Needs**	39	16	24	27	54	66	The increase reflects the heightened use of virtual learning platforms both during and after the COVID-19 pandemic.

**Internet**	21	3	N/A	25	24	13	The fluctuation of the data suggests the better overall access to internet services in later years.

**Child Life Specialist Referral**	N/A	25	0	12	2	On-site assistance	The varying data could indicate that the child life team is addressing these needs through regular interactions with families.

**Assistance With Clothing/Uniform**	N/A	79	72	N/A	82	104	The data show that there are shifting demands for this support; however, the need is consistent.

**E-Reader**	N/A	N/A	96	115	125	None available	The increase in data over time highlights the growing importance of providing devices to support educational and recreational activities during treatment.


While the total number of families screened declined slightly after 2023, this reflected the transition to more intensive, individualized interventions following the integration of school coordinators. Supporting an average of 90 patients per month, each coordinator facilitated homebound instruction, hospital school enrollment, and reintegration planning for approximately 1,080 patients annually, in alignment with state guidance on General Education Homebound services and instructional eligibility criteria (Texas Education Agency, 2021). An average of 40 new patients per year were enrolled in the hospital-based school program, maintaining consistent academic engagement during treatment.

Fluctuations were also observed in other support categories, including internet access, clothing assistance, and referrals to Child Life services. Child Life services are delivered by trained specialists who support children in coping with illness and medical treatment through age-appropriate education and therapeutic medical play. These fluctuations reflect shifting social and educational needs as pandemic-related challenges eased and community-based resources became more readily available. Additionally, the decline in direct social work referrals between 2021 and 2024 suggests that earlier screening and strengthened cross-disciplinary communication reduced crisis-level needs by proactively addressing educational and psychosocial concerns.

## Programmatic and Qualitative Outcomes

Qualitative feedback gathered from families and staff emphasized several key outcomes aligned with program aims:

1. Increased Educational Preparedness:
  Families reported feeling more organized and equipped for the start of the school year. The combination of supplies, technology, and individualized academic support reduced stress and improved confidence in being able to manage school transitions during treatment.
2. Improved Communication and Collaboration:
  The integration of school coordinators in 2023 enhanced communication between families, healthcare teams, and educational institutions. Coordinators facilitated direct contact with teachers, school nurses, and district liaisons, ensuring continuity of education.
3. Enhanced Continuity of Schooling:
  Annual screenings allowed the psychosocial team to identify educational gaps early and implement tailored interventions, including homebound or hybrid learning options. This structured approach enabled consistent educational engagement despite frequent medical absences.
4. Resource Accessibility and Equity:
  Families noted improved access to essential community resources, including food assistance, internet connectivity, and low-cost dental and vision care. Materials were provided in both English and Spanish, improving accessibility for diverse populations and promoting equity in service delivery.

## Expansion Through the 2025 Back-to-School Program

Building on earlier program successes, the August 2025 Back-to-School Program expanded both reach and evaluation efforts. Nearly 140 families participated in a one-day, multidisciplinary event informed by Social Drivers of Health (SDOH) data, non-medical factors such as family income, housing stability, access to education, transportation, and internet access that influence health, treatment adherence, and school engagement. Supports provided included 359 backpacks with school supplies, 53 new public library cards, and technology referrals for more than 60 families.

Families received individualized assistance from school coordinators, social workers, and child life specialists, allowing for comprehensive assessment and support. Before leaving the event, families completed post-event surveys assessing satisfaction, preparedness, and perceived stress. More than 90% of participants reported feeling “well prepared” or “very well prepared” for the upcoming school year, citing improved communication with schools and reduced financial and logistical stress (Table 3).

**Table 3:** Outcomes and Interpretation of the 2025 In-Person Back-to-School Event.


	KEY FINDINGS	INTERPRETATION

**Participation**	200 families registered 138 attended (51 hematology, 87 oncology)	High turnout validates targeted outreach to vulnerable populations

**Backpacks/School Supplies Provided**	359 backpacks distributed at event 50 delivered to admitted patients in hospital	Immediate tangible support reduced material barriers to school readiness

**Technology Support**	66 families reported device needs 53 new families signed up for public library cards	Addressing digital inequities supports academic continuity and long-term engagement with educational resources

**Community Engagement**	17 community partners participated on-site	Strong community integration expanded family access to ongoing educational and social resources

**Family Impact**	100% of surveyed families felt more prepared for school 98% found event convenient	Families reported high satisfaction and perceived readiness, highlighting event’s effectiveness and relevance

**Partner Impact**	13 organizations rated event highly for coordination, logistics, and engagement.	Strong partner satisfaction ensures sustainability and scalability of future practice-based initiatives


Qualitative feedback from families and staff highlighted that direct access to educational personnel and tangible supports reduced stress and promoted a sense of normalcy. These findings demonstrate the program’s ability to integrate educational resources with psychosocial care and support a sustainable, proactive model that can be replicated annually or adapted for other chronic illness populations.

## Summary of Key Outcomes

As illustrated in Table 2, between 2020 and 2024, the Back-to-School Screening Program demonstrated sustained growth and impact in supporting children with cancer and blood disorders and their families. Specifically, over this period, 1,193 families were screened, with increasing access to school supplies, technology, and coordinated educational supports. The integration of school coordinators in 2023 enhanced the depth and efficiency of school-related interventions, contributing to consistent annual reach in school reintegration services and hospital-based school enrollment. Families reported improved preparedness and satisfaction, citing stronger collaboration and access to resources. The program’s successful expansion in 2025 further reinforced these outcomes, extending support to 140 families and generating measurable improvements in educational readiness.

## Link to Program Aims

The results of the Back-to-School Program support the program’s original aims, demonstrating strong implementation fidelity and measurable outcomes across both quantitative and qualitative measures. Structured screening helped identify and address academic and technology-related barriers for children with cancer and blood disorders, while improved communication among families, healthcare teams, and schools strengthened coordination of care. The program supported continuity of learning throughout treatment and recovery and promoted more equitable access to educational and community resources. Through targeted screening, timely intervention, and ongoing collaboration, the program has become an integral component of the institution’s psychosocial care model.

## Discussion/Lessons Learned

The implementation and subsequent evaluation of the Back-to-School Screening Program and along with the recently added 2025 Back-to-School initiative demonstrate meaningful progress toward the original program aims of improving coordination between healthcare and educational systems, identifying barriers to academic reentry, and connecting families with supportive resources. By systematically screening children with cancer and blood disorders for their educational needs, providing school supplies, and linking families to on-site community partners, this practice-based initiative strengthened school reintegration for children. Families reported feeling supported and more prepared for school, while staff noted that the structured approach allowed them to identify and assess needs proactively.

## Connections to Aims and Prior Literature

Consistent with prior work in coordinated care and psychosocial oncology, including family-centered integration frameworks within the *Continuity in Education* (CIE) literature (Delloso et al., 2021), our findings indicate that early identification of academic barriers through standardized screening enhances collaboration among hospital-based school coordinators, social workers, and educators. The model aligns with existing evidence showing that multidisciplinary educational interventions reduce school absences and stress for children undergoing cancer treatment, while also supporting caregivers in navigating complex educational systems (Delloso et al., 2021; Emerson et al., 2016). The program complements existing recommendations by explicitly linking families to tangible educational resources and technology, addressing social determinants of learning, and facilitating structure communication between multiple stakeholders.

## Methodological Clarification

The initiative was implemented as an intervention using descriptive and process evaluation methods. Families and staff participated by completing structured screening tools, informal interviews, and brief post-event surveys. The internally developed screening tool (Appendix 2), reviewed by the multidisciplinary team, assessed academic needs, technology access, and psychosocial support. Data included family needs, staff observations related to school re-entry, and measures of family satisfaction and preparedness. Quantitative data were summarized descriptively, and qualitative feedback was analyzed to identify recurring needs and gaps. All procedures followed hospital policies, with verbal informed consent obtained from participating families.

## Limitations

Despite the positive outcomes, several operational challenges limited full program reach and fidelity. High patient volumes and staffing shortages, especially during and after the COVID-19 pandemic, restricted consistent administration of the screening tool. Further, the prioritization of active oncology patients limited outreach to off-therapy or non-oncology populations, creating potential gaps in continuity of educational support. Additionally, the variability in community partners’ eligibility criteria and the incomplete screening rate suggest that some families with significant academic needs were missed. Time constraints also impacted both the depth of screening and the follow-up process.

## Implications for Practice and Future Replication

The program’s success underscores the feasibility and value of a “one-stop” model that integrates academic, psychosocial, and community resources. Lessons learned from implementation highlight several practical considerations for replication, including expanding technology access through community partnerships (e.g., Hopecam and public libraries), broadening screening coverage to include off-therapy and other chronic illness populations, and incorporating ongoing fidelity checks to ensure consistency in screening implementation and follow-up.

## Empirical Insights

Staff and family feedback reinforced the program’s emotional and practical impact Families expressed feeling “seen and supported,” while coordinators emphasized that the practice-based initiative “gave us the structure to identify needs before school starts.” This underscores the importance of embedding educational advocacy within pediatric healthcare settings to promote equity and continuity in learning.

## Synthesis of Key Takeaways

Overall, the Back-to-School Screening Program and the 2025 Back-to-School initiative illustrate how integrated healthcare and education partnerships can improve patient and family outcomes. The program demonstrates high feasibility, strong family engagement, and alignment with national CIE principles. While staffing constraints and incomplete date limitations remain, this program highlights actionable strategies for improving academic continuity for children with chronic illness.

## Conclusion

The Back-to-School Screening Program and the 2025 Back-to-School initiative demonstrate that structured, multidisciplinary collaboration among healthcare, education, and community partners can significantly enhance academic and psychosocial outcomes for children with cancer and blood disorders. These initiatives successfully identified educational barriers early, provided school readiness materials and individualized supports, and facilitated smoother transitions back to the classroom. Collectively, the findings affirm that proactive, systematic screening, integrated within psychosocial care, yields measurable benefits for both patients and families.

While the program has strengthened cross-sector communication and resource coordination, ongoing challenges remain in sustaining long-term follow-up, expanding access across diverse populations, and aligning data systems between healthcare and educational institutions. Future refinements should continue to evaluate the program’s scalability and explore strategies for embedding educational advocacy within broader models of chronic illness care.

For practitioners and policymakers, this model offers a replicable framework for integrating educational assessment and intervention into pediatric healthcare systems, bridging medical and academic domains to promote resilience, equity, and lifelong learning for children affected by serious illness.

## Additional Files

The additional files for this article can be found as follows:

10.5334/cie.288.s1 Supplementary File 1. Appendix 1 is the timeline for the Back-to-School Screening Project from 2020 to 2025.

10.5334/cie.288.s2 Supplementary File 2. Appendix 2 is the 2025 Form used for the Back-to-School Screener (guardian completed).
