# Supplementary material for: Back-to-School Screening for Children with Cancer and Hematologic Disorders: Bridging Healthcare and Education
Source: Contin Educ. 2026 Jan 29;7(1):1–11. doi: 10.5334/cie.288 (PMC12857629; doi:10.5334/cie.288)
Supplement: Supplementary File 1. — Appendix 1 is the timeline for the Back-to-School Screening Project from 2020 to 2025. [file cie-7-1-288-s1.pdf]

Back-to-School Screening for Children With Cancer and Hematologic Disorders: Bridging Healthcare and Education

Appendix 1:Timeline For School Screening Project from 2020-2025

Fritsch, Matthews, Kara, Deeter

Timeline For School Screening Project from 2020-2025

2020: The Start of the COVID-19 Pandemic & School Closures

- **March 2020**: Schools closed worldwide due to the COVID-19 pandemic. In the state, school districts extended spring breaks and began planning for remote learning contingencies.
- **April 17, 2020**: The state governor announced the closure of schools for the remainder of the academic year, and districts began implementing virtual/alternative learning formats.
- **June 18, 2020**: The state Commissioner of Education announced that schools would open in the fall.
- **June 2020**: **Social Worker Outreach**: Social workers contacted school districts in the large metropolitan area and surrounding counties to understand and communicate their remote learning plans.
- **June 2020**: **Spreadsheet Development**: A tracking spreadsheet was created to monitor school district plans, including learning formats (virtual, onsite, hybrid), availability of hotspots, computers, and school calendar dates.
- **March – October 2020**: **Clinic Outreach**: Health screenings were conducted in outpatient clinics, adding school-related questions to identify educational needs.
- **August 2020**: **School Supplies Distribution**: Distribution of school supplies to children and families to support remote learning during the pandemic, completed at the start of the 2020-2021 school year.

2021: Expanding the Program and Adapting to Continued Uncertainty

- **August 2021**: **Ongoing School Closures**: Many schools remained closed or operated in hybrid/virtual formats, with the state education agency providing operational flexibility to local school systems.
- **2021-2022 School Year**: **Instructional Flexibility**: Schools were required to offer both remote and in-person classes, including synchronous (real-time) and asynchronous (self-paced) instruction options.
- **June 2021**: **Updated Spreadsheet**: The spreadsheet expanded to include services like free breakfast and lunch for students in need.
- **July 2021**: **Increased Collaboration with Community Resources**: Partnerships with local charities provided additional services, including free clothing for children who had outgrown their old clothes.
- **August 2021**: **Continued School Supplies Distribution**: Ongoing distribution of school supplies to inpatients and outpatients.
- **Intake Form**: A one-page paper intake form was posted throughout the clinic for families to identify any school-related needs, such as technology or school supplies.

This document contains supplementary material for the above-mentioned article, as provided by the authors.
The original article can be downloaded from https://doi.org/10.5334/cie.288

September 2022: Continued Support and Expansion of Services

- **Program Consolidation**: The school reentry program continued to serve families, focusing on maintaining communication about evolving school plans and providing resources.
- **Expanded Community Outreach**: Increased efforts to connect with schools and community organizations to ensure children with cancer and their families had the necessary resources, particularly as schools returned to 100% in-person attendance.

January 2023: Patient Educational and Vocational Services Established

- A comprehensive program was created, providing hospital-based school instruction, school advocacy, and academic services for the oncology patients.
- **Neuropsychological Evaluation**: Integrated neuropsychological evaluations were incorporated to address the cognitive effects of cancer therapy on school re-entry.

2023: Full School Reentry & Enhanced Services

- **Reentry Support for Returning Students**: Focus on helping children who had been away from school transition back, particularly those who had experienced significant physical growth during the pandemic.
- **Clothing Assistance**: A formalized collaboration with local charities provided new clothing to children who had outgrown their previous garments.
- **Ongoing Support for Virtual Learners**: Continued support was offered to students learning virtually or through hybrid models, including updated resources and tools.
- **Intake Form Continued**: The intake form remained in use, with School Coordinators engaging more than 60 patients per month between March and September, serving over 360 patients.
- **Expanded Advocacy**: Two outpatient School Coordinators focused on advocacy and school reentry support, assessing needs and providing appropriate resources for patients and families.

2024: Digital Transformation and Streamlined Services

- **Shift to QR Code Intake**: The paper intake form was replaced with a QR code system, allowing parents to access the form via Microsoft 365 and complete it easily from their phones as they arrived at the clinic.
- **Digital Form Enhancements**: The digital form included updated psychosocial questions to provide more specific support from child life, social work, and school coordinators.
- **Ongoing Program Support**: Continued support was provided to both in-person and virtual learners, ensuring families had access to school supplies, food, technology, and other resources.
- **Increased Patient Reach**: The program expanded to serve an average of 400 patients annually, providing comprehensive support for school reentry and advocacy.

2025: First Annual Back-to-School Initiative

- **Event Launch:** Inaugural one-day, one-stop event designed to support school re-entry for children with cancer and blood disorders.
- **Targeted Outreach:** Families identified through social determinants of health (SDOH) data to ensure support reached those most in need.

- **Resource Distribution:** Provided backpacks, school supplies, clothing assistance, and technology resources; included device access support through Hopecam, Houston Public Library programs, and individualized social work interventions.
- **Community Engagement:** 17 community partners participated on-site, helping families connect with educational and social resources. The local Ronald McDonald House attend the event and we provided them with 100 backpacks with supplies to provide to families who were unable to attend the event in person.
- **Family Impact:** Families reported feeling more prepared for school and rated the event convenient; most valued resources included tangible supplies and school coordinator support.
- **Partner Impact:** Organizations rated the initiative highly for coordination, logistics, and family engagement; all expressed interest in future participation.
- **Program Growth:** Needs identified (clothing, eyeglasses, internet access) are being addressed through ongoing collaboration with social work and community partners, positioning the initiative as both an immediate intervention and a platform for continued program development.
