# Supplementary material for: Back-to-School Screening for Children with Cancer and Hematologic Disorders: Bridging Healthcare and Education
Source: Contin Educ. 2026 Jan 29;7(1):1–11. doi: 10.5334/cie.288 (PMC12857629; doi:10.5334/cie.288)
Supplement: Supplementary File 2. — Appendix 2 is the 2025 Form used for the Back-to-School Screener (guardian completed). [file cie-7-1-288-s2.pdf]

Back-to-School Screening for Children With Cancer and Hematologic Disorders: Bridging Healthcare and Education

Appendix 2: 2025 Form used for the Back To School Screener (guardian completed)

Fritsch, Matthews, Kara, Deeter

2025 Form used for the Back To School Screener (guardian completed)

Patient and Caregiver Information

1. Patient Last Name
2. Patient First Name
3. Patient Date of Birth (MM/DD/YYYY)
4. Caregiver First and Last Name
5. Relationship to Patient
6. Caregiver Contact Number
7. Caregiver Email Address
8. Patient Primary Diagnosis (oncology, hematology)

Computer

9. Does your child need a tablet or laptop for educational purposes?

Internet

10. Does your child have access to internet at home?

School Supply Needs (Backpacks and School Supplies will be provided at the event for all school age children in your home)

11. How many total school age children live in your home?
12. Select the number of backpacks needed for each age group below.
    a. Preschool
    b. Elementary (K – 5)
    c. Middle/High School (6 – 12)
13. Do your children need clothing for school?

School Information

14. Would you like to talk with a School Coordinator at the event to discuss school questions/concerns?
15. What type of school does your child attend?
    a. Public
    b. Private
    c. Homebound
    d. Home-schooled
16. School District
17. School Name

This document contains supplementary material for the above-mentioned article, as provided by the authors.

The original article can be downloaded from https://doi.org/10.5334/cie.288

18. Grade in School
19. Does your child receive school services, accommodations or supports?
    a. Special Education (IEP/ARD)
    b. 504 Plan
    c. Not Sure
    d. None
20. Do you or your child have any of the following worries or fears regarding returning back to school? Please check all that apply. Choose "Other" to list additional worries/fears.
    a. No concerns
    b. Bullying
    c. Academic Concerns
    d. Hair Loss
    e. Physical Limitations
    f. Social/Emotional Concerns
    g. Change in Routine/Attendance
    h. Coping Concerns with School Reentry
21. Please list any other concerns, questions or resources needed.

Transportation

22. What form of transportation will you use to get to the event
    a. Car (free parking)
    b. Public Transport
    c. I have transportation concerns for this event
